# Monoclonal antibodies for differentiating infections of three serological-related tospoviruses prevalent in Southwestern China

**DOI:** 10.1186/s12985-016-0525-3

**Published:** 2016-04-27

**Authors:** Yu-Han Chen, Jiahong Dong, Wan-Chu Chien, Kuanyu Zheng, Kuo Wu, Shyi-Dong Yeh, Jing-Hua Sun, Yun-Chi Wang, Tsung-Chi Chen

**Affiliations:** Department of Biotechnology, Asia University, Wufeng Taichung, 41354 Taiwan; Yunnan Provincial Key Laboratory of Agricultural Biotechnology, Key Laboratory of Southwestern Crop Gene Resources and Germplasm Innovation of Ministry of Agriculture, Biotechnology and Germplasm Resources Institute, Yunnan Academy of Agricultural Sciences, Kunming, 650223 China; Department of Plant Pathology, National Chung Hsing University, Taichung, 40227 Taiwan; NCHU-UCD Plant and Food Biotechnology Center, National Chung Hsing University, Taichung, 40227 Taiwan; Department of Medical Research, China Medical University Hospital, China Medical University, Taichung, 40402 Taiwan

**Keywords:** Tospovirus, Monoclonal antibody, Serological assay, Diagnosis, Quarantine

## Abstract

**Background:**

The thrips-borne tospoviruses Calla lily chlorotic spot virus (CCSV), Tomato zonate spot virus (TZSV) and a new species provisionally named Tomato necrotic spot associated virus (TNSaV) infect similar crops in southwestern China. The symptoms exhibiting on virus-infected crops are similar, which is difficult for distinguishing virus species by symptomatology. The sequences of nucleocapsid proteins (NPs) of CCSV, TNSaV and TZSV share high degrees of amino acid identity with each other, and their serological relationship was currently demonstrated from the responses of the previously reported monoclonal antibodies (MAbs) against the NP of CCSV (MAb-CCSV-NP) and the nonstructural NSs protein of *Watermelon silver mottle virus* (WSMoV) (MAb-WNSs). Therefore, the production of virus-specific antibodies for identification of CCSV, TNSaV and TZSV is demanded to improve field surveys.

**Methods:**

The NP of TZSV-13YV639 isolated from *Crinum asiaticum* in Yunnan Province, China was bacterially expressed and purified for producing MAbs. Indirect enzyme-linked immunosorbent assay (ELISA) and immunoblotting were conducted to test the serological response of MAbs to 18 tospovirus species. Additionally, the virus-specific primers were designed to verify the identity of CCSV, TNSaV and TZSV in one-step reverse transcription-polymerase chain reaction (RT-PCR).

**Results:**

Two MAbs, denoted MAb-TZSV-NP(S15) and MAb-TZSV-NP(S18), were screened for test. MAb-TZSV-NP(S15) reacted with CCSV and TZSV while MAb-TZSV-NP(S18) reacted specifically to TZSV in both indirect ELISA and immunoblotting. Both MAbs can be used to detect TZSV in field-collected plant samples. The epitope of MAb-TZSV-NP(S18) was further identified consisting of amino acids 78–86 (HKIVASGAD) of the TZSV-13YV639 NP that is a highly conserved region among known TZSV isolates but is distinct from TNSaV and TZSV.

**Conclusions:**

In this study, two MAbs targeting to different portions of the TZSV NP were obtained. Unlike MAb-CCSV-NP reacted with TNSaV as well as CCSV and TZSV, both TZSV MAbs can be used to differentiate CCSV, TNSaV and TZSV. The identity of CCSV, TNSaV and TZSV was proven by individual virus-specific primer pairs to indicate the correctness of serological responses. We also proposed an serological detection platform using MAb-CCSV-NP, MAb-TZSV-NP(S15) and MAb-TZSV-NP(S18) to allow researchers and quarantine staff to efficiently diagnose the infections of CCSV, TNSaV and TZSV in China and other countries.

**Electronic supplementary material:**

The online version of this article (doi:10.1186/s12985-016-0525-3) contains supplementary material, which is available to authorized users.

## Background

The viruses of *Tospovirus*, the only plant-infecting genus in the family *Bunyaviridae*, cause severe damage to several agricultural crops worldwide [1, 2]. Tospoviruses have enveloped quasi-spherical particles that are 80–120 nm in diameter, and a tripartite-segmented single-stranded RNA (ssRNA) genome [3]. The large (L) RNA is in negative sense and contains one single open reading frame (ORF) encoding an RNA-dependent RNA polymerase for replication and transcription [4, 5]. Both middle (M) and small (S) RNAs are ambisense, each consisting of two bi-directional ORFs flanked by an AU-rich intergenic region. The M RNA encodes a movement protein (NSm) from the viral sense and the envelope glycoproteins Gn and Gc from the viral complementary sense [6, 7]. The S RNA encodes a suppressor of plant gene silencing (NSs) from the viral sense and an RNA-encapsidating nucleocapsid protein (NP) from the viral-complementary sense [8–10].

According to the International Committee on Taxonomy of Viruses, the criteria for demarcating a tospovirus species include thrips-vector specificity, specific host range, serology of NP, and lower than 90 % amino acid identity of the NP [3]. Tospoviruses can be serologically grouped with the aid of antisera against the NPs. The original demarcation consisted of four serogroups (I to IV) with six known tospoviruses [11]. However, a type member-based serological classification system was recommended as increasing characterized tospoviruses [12]. Currently, a more comprehensive serological grouping has been established through experimental evidence. Most of the known tospoviruses are now classified into four serogroups using *Groundnut yellow spot virus* (GYSV), *Iris yellow spot virus* (IYSV), *Tomato spotted wilt virus* (TSWV) and *Watermelon silver mottle virus* (WSMoV) as type members [13–15]. The serological grouping of tospoviruses matches well with their phylogenetic clustering, in which tospoviruses sharing more than 51.8 % similarity at the NP amino acid sequence level are serologically related [13, 16]. Because of the high degree of sequence identity within the same serogroup, distinguishing and diagnosing tospoviruses rely on monoclonal antibodies (MAbs) with a higher specificity to a particular species. However, tospoviruses, such as Capsicum chlorosis virus (CaCV), *Groundnut bud necrosis virus* (GBNV), *Watermelon bud necrosis virus* (WBNV) and WSMoV, sharing 80 % or higher NP amino acid sequence similarity are still difficult to distinguish even when MAbs are used [17]. Therefore, when generating MAbs, it is critical to validate the serological assays to prevent false diagnosis.

Tospoviruses are causing significant losses in yield and quality of several economic crops in China [18, 19]. Two new tospoviruses Tomato necrotic spot associated virus (TNSaV) and Tomato zonate spot virus (TZSV) infecting tomato were first discovered in Guizhou and Yunnan provinces, respectively [19, 20]. The serological relationship between TNSaV and TZSV was demonstrated by the cross reaction with the antiserum against the TZSV NP [19]. TZSV currently becomes the important threat infecting tomato, tobacco and ornamentals in southwestern China, and *Frankliniella occidentalis* (Pergande) is its main transmissible vector [18, 20–22]. Calla lily chlorotic spot virus (CCSV), first collected from calla lily in Taiwan, is occurring in Yunnan Province that infects tobacco and spider lily [23, 24]. The transmissible vector of CCSV and TNSaV in China remains unknown.

Symptomatology is insufficient for identification of virus species due to the fact that similar symptoms on the same crop may be caused by different tospoviruses. Indeed, both TNSaV and TZSV induce yellow and necrotic ringspots on tomato fruits [19, 20] and all of CCSV, TNSaV and TZSV cause chlorotic and necrotic spots on tobacco leaves [19, 21, 24]. The NPs of CCSV, TNSaV and TZSV share high degrees of amino acid identity (80.9–85.8 %) with each other [19, 20, 23], and their serological relationship was recently demonstrated through the serological assays using the MAbs against the NP of CCSV (MAb-CCSV-NP) [25] and the NSs protein of WSMoV (MAb-WNSs) [26]. Although the virus-specific primers for reverse transcription-polymerase chain reaction (RT-PCR) can be used to identify tospovirus species when antibodies are unavailable or indistinguishable, the need of professional skill and equipment and the cost of manpower and time limit the application of RT-PCR for a large amount of samples in epidemiological investigation. Enzyme-linked immunosorbent assay (ELISA) is an efficient serological method for field survey of viruses, and the titer and specificity of antibodies are very important for successful assays. CCSV, TNSaV and TZSV induce similar symptoms on their common natural hosts in southwestern China [19, 24], the production of virus-specific antibodies for identification of these tospoviruses is essential to improve field surveys.

In this study, MAbs against the NP of the TZSV isolate 13YV639, which was collected from spider lily (*Crinum asiaticum* L.) in Yunnan Province, China, were screened. Two MAbs with distinct serological reactivity were obtained. Using the newly generated MAbs and previously reported MAbs, we proposed an efficient serological assay to differentiate CCSV, TNSaV and TZSV in field samples.

## Methods

### Virus sources and maintenance

The isolates of TZSV-13YV639 and Hippeastrum chlorotic ringspot virus (HCRV)-13YV640 [27] were collected from *C. asiaticum* and *Hymenocallis littoralis* (Jacq.) Salisb., respectively, in Yunnan Province, China. TNSaV-2009-GZT was collected from tomato in China [19]. CCSV-TW isolated from calla lily [23], Groundnut chlorotic fan-spot virus (GCFSV)-PD2 from groundnut [28], Melon yellow spot virus (MYSV)-TW from watermelon [29], WSMoV-DD6 from watermelon, Capsicum chlorosis virus (CaCV)-V1 from orchid, and TSWV-Z from calla lily [30] were collected in Taiwan. Both *Groundnut ringspot virus* (GRSV)-BR [31] and *Tomato chlorotic spot virus* (TCSV)-BR-03 isolated from tomato were collected in Brazil [32]. *Impatiens necrotic spot virus* (INSV)-M was collected from impatiens in the United States [33]. IYSV was collected from iris in the Netherlands [34]. Tomato yellow ring virus (TYRV)-t was collected from tomato in Iran [35]. Alstroemeria necrotic streak virus (ANSV) was collected from *Alstroemeria* sp. in Colombia [36]. *Groundnut bud necrosis virus* (GBNV)-To isolated from tomato and *Watermelon bud necrosis virus* (WBNV)-JT from watermelon were collected in India [37]. Chrysanthemum stem necrosis virus (CSNV)-TcCh07A was isolated from chrysanthemum in Japan [15]. All tospoviruses were maintained in the systemic host *Nicotiana benthamiana* Domin and the local lesion host *Chenopodium quinoa* Willd. by mechanical inoculation. The inocula were prepared by grinding the virus-infected leaf tissue in 10 mM potassium phosphate buffer (pH 7.0) containing 0.1 % sodium sulfite. The inoculated plants were kept in a temperature-controlled isolation greenhouse (26–28 °C).

### Construction of NP ORF into pET-28b(+) vector

Total RNA from 100 mg of leaf tissue of a TZSV-13YV639-infected *N. benthamiana* plant was extracted using the Plant Total RNA Miniprep Purification Kit (GMbiolab, Taichung, Taiwan). The NP ORF of TZSV-13YV639 was amplified with the primers TZN-NcoI and TZN-Xho-Kpnc (Additional file 1: Table S1) using the One-Step RT-PCR Kit (GMbiolab). Ten microgram of total RNA, 200 nM individual primers, 25 U One-Step RT-PCR enzyme mix, 1/5 volume of reaction buffer and 1/5 volume of enhancer buffer (GMbiolab) were mixed for one-step RT-PCR amplification. Synthesis of cDNA was conducted at 50 °C for 30 min, and inactivation at 94 °C for 2 min; PCR was performed by 35 cycles of strand separation at 94 °C for 1 min, annealing at 60 °C for 30 s and synthesis at 72 °C for 1 min; and a final reaction at 72 °C for 7 min. The amplicon was cloned into the TOPO TA cloning vector pCR2.1-TOPO (Invitrogen, Carlsbad, CA) to obtain the recombinant plasmid pTOPO-TZSV-NP. The nucleotide sequence of the amplicon was verified by the Mission Biotech Company (Taipei, Taiwan) using ABI3730 XL DNA Analyzer (Perkin-Elmer Applied Biosysterms, Foster City, CA). The DNA fragment corresponding to the NP ORF of TZSV-13YV639 was released from pTOPO-TZSV-NP using the *Nco*I and *Xho*I restriction enzymes, and ligated with the pET-28b(+) vector (Novagen, Madison, WI) treated with the same restriction enzymes. The resulting recombinant plasmid pET-TZSV-NP was transformed into *E. coil* DH5α for cloning. Subsequently, the plasmid was isolated from *E. coil* DH5α and transferred into *E. coil* Rosetta BL21(DE3) (Novagen) for protein expression.

### Expression and purification of recombinant NP (rNP)

One milliliter of bacterial overnight culture was added to aliquots of 100 ml LB medium containing kanamycin (50 μg/μl) and chloramphenicol (34 μg/μl), then incubated at 37 °C for 2 h with shaking at 225 rpm (the cell number reaches OD_600_ = 1.0). Protein expression was induced by the addition of 1 mM IPTG. Three hours after induction, bacterial cells were collected by centrifugation at 8,000 rpm at 4 °C for 10 min. The pellet was resuspended in 8 ml native purification buffer (50 mM NaH_2_PO_4_, pH 8, and 0.5 M NaCl) containing 1 mg/ml lysozyme, and incubated on ice for 30 min. The solution was sonicated on ice using a sonicator equipped with a microtip for six 10-s bursts at high intensity and a 10-s cooling period between each burst. After centrifugation at 8,000 rpm at 4 °C for 15 min, the pellet was resuspended in 8 ml denaturing binding buffer (8 M urea, 20 mM sodium phosphate, pH 7.8, and 0.5 M NaCl) and incubated at room temperature for 30 min. The pellet suspension was diluted by half with protein sample buffer (12.5 mM Tris-HCl, pH 6.8, 10 % glycerol, 2 % SDS, 2 % β -mercaptoethanol and 0.001 % bromphenol blue), boiled for 3 min, and put on ice for 1 min. The isolated rNP was verified by 12 % SDS-PAGE and immunoblotting using rabbit antiserum against the histidine tag (RAs-His) (Viogene, Taipei, Taiwan) and mouse MAb against the NP of CCSV (MAb-CCSV-NP) [25].

The rNP was purified and separated in 12 % SDS-PAGE, visualized by soaking the gel in cold 0.3 M KCl to excise the part of rNP-containing gel. The rNP was further eluted from the polyacrylamide gel using a Model 442 Electro-Eluter (Bio-Rad, Hercules, CA). Yields of the purified rNP were estimated using Spot Density of AlphaInnotech IS2000 (AlphaInnotech Corporation, San Leandro, CA) comparing the sample with the quantified bovine serum albumin.

### Preparation of MAb

The 8-week-old BALB/cByJ female mice were first immunized with 50 μg of the purified rNP (in 250 μl of PBS) emulsified with equal volume of Freund’s complete adjuvant (Difco Laboratories, BD, Franklin Lakes, NJ) by intraperitoneal injection. Mice were then injected on a weekly basis for two more weeks using 50 μg of rNP emulsified with Freund’s incomplete adjuvant (Difco Laboratories). Mice were injected a fourth time without adding the adjuvant, then sacrificed 3 days after the last injection to harvest splenocytes. The splenocytes were fused with Spll/0-ag/14 myeloma as described previously [38]. Hybridoma cells secreting anti-rNP antibodies in cultural media were screened by indirect ELISA using the crude leaf sap of *N. benthamiana* infected with TZSV-13YV639 as the antigen. Subsequently, the selected antibody-secreting hybridoma cells were cloned by limiting dilution method. Antibodies were further produced in ascitic fluids by intraperitoneal injection of 10^6^ hybridoma cells into Pristane-primed BALB/cByJ female mice.

### Immunoblotting

*N. benthamiana* leaves infected with virus were ground in protein sample buffer at a 1/50 dilution for immunoblot analysis. Lysates were boiled for 3 min, placed on ice for 1 min and centrifuged at 13,300 rpm for 3 min. Supernatant was collected and separated by 12 % SDS-PAGE, then transferred onto nitrocellulose (NC) membranes in transfer buffer (25 mM Tris, 192 mM glycine and 20 % methanol) embedded in ice by VE-186 Mini Blotting Electrophoresis Cell (TANON, Shanghai, China) at 120 V for 30 min. Transfer of proteins was visualized by a Ponceau S stain, and the NC membranes were washed with TSW buffer (10 mM Tris–HCl, pH 7.4, 154 mM NaCl, 0.25 % gelatin, 0.1 % Triton X-100 and 0.02 % SDS), then incubated with primary antibodies diluted in TSW buffer for 30 min. RAs-His (Viogene) was used at a 10^−3^ dilution, and MAb-CCSV-NP [25] was used at a 10^−4^ dilution. After washing, the NC membranes were incubated with alkaline phosphatase (AP)-conjugate goat anti-rabbit IgG or goat anti-mouse IgG (1/5000; Jackson Immuno Research Laboratories, Inc.) in TSW buffer for 30 min. Color was developed by adding 50 μl of 50 mg/ml nitro blue tetrazolium chloride (NBT) and 25 μl of 50 mg/ml 5-bromo-4-chloro-3-indoyl phosphate (BCIP) in 7.5 ml substrate buffer (100 mM Tris–HCl, pH 9.5, 100 mM NaCl and 5 mM MgCl_2_). Lastly, NC membranes were submerged in water to terminate the reaction.

### Indirect ELISA

Indirect ELISA was conducted according to a previously method [39] with modifications as below. Polystyrene microtitration plates with 200 μl per well of the crude extract of virus-infected plant tissues at a 1/50 dilution or the different concentrations of rNP diluted within coating buffer (15 mM Na_2_CO_3_, 35 mM NaHCO_3_ and 3 mM NaN_3_, pH 9.6) were incubated at 37 °C for 50 min, then washed with PBST buffer (137 mM NaCl, 1 mM KH_2_PO_4_, 8 mM Na_2_HPO_4_‧12H_2_O, 3 mM KCl, 3 mM NaN_3_ and 0.05 % Tween 20). The MAbs were diluted in conjugate buffer (PBST buffer containing 2 % PVP-40 and 0.2 % ovalbumin), and were loaded to the plates (200 μl for each well). The plates were incubated at 37 °C for 50 min and then washed with PBST buffer. Each well was loaded with 200 μl of the secondary antibody AP-conjugate goat anti-mouse IgG (1/5000; Jackson Immuno Research Laboratories, Inc.) diluted in conjugate buffer. The plates were incubated at 37 °C for 50 min, and then washed with PBST buffer. Color-developing solution was prepared by dissolving ρ -nitrophenyl phosphate disodium hexahydrate (GMbiolab) in colorization buffer (9.7 % diethanolamine and 3 mM NaN_3_) to a final concentration of 1 mg/ml, and 180 μl of solution was loaded to each well. Twenty to sixty min after the addition of the enzyme substrate, plates were placed in Model 680 microplate reader (Bio-Rad) to measure absorbance at 405 nm (A_405_).

### Sequence analysis

NP sequences of CCSV-TW (AY867502), TNSaV-2009-GZT (KM355773), TZSV-13YV639 (KP684519) and TZSV-Tomato-YN (NC_010489) were obtained from GenBank, and were aligned using ClustalW (http://workbench.sdsc.edu/). Sequence comparisons of NPs from different TZSV isolates were conducted using BLASTP (NCBI; http://blast.ncbi.nlm.nih.gov/Blast.cgi).

### Epitope mapping

To construct a pET-28b(+)-based plasmid pET28-eGFP for expressing enhanced GFP (eGFP) as a tag, the eGFP ORF was amplified by PCR using the primers eGFP-EcoR-f (5′-G*GAATTC*ATGGTGAGCAAGGGCGAGGAG-3′) and eGFP-Xho-r (5′-G*CTCGAG*TTACTTGTACAGCTCGTCCAT-3′), containing the *Eco*RI and *Xho*I recognition sites (italic), respectively. The pET-28b(+) vector was digested with *Eco*RI/*Xho*I to insert the eGFP ORF. DNA fragments corresponding to different portions of the NP ORF of TZSV-13YV639 were amplified by PCR using pTOPO-TZSV-NP as the template. The primers used for amplifications are shown in Additional file 1: Table S1. PCR was performed using the same conditions as NP ORF. The amplicons were cloned into the pCR2.1-TOPO vector (Invitrogen) to verify the sequence, and subsequently released by digestion with *Nco*I/*Xho*I to construct into pET-28b(+) or digestion with *Nco*I/*Eco*RI to construct into pET28-eGFP treated with the same restriction enzymes. Expression of the recombinant eGFP was verified by rabbit antiserum RAs-GFP at a 1/5000 dilution [40] in immunoblotting as described above.

### Primer design for virus detection

The virus-specific primer pairs CC-f/CC-r, TN-f/TN-r and TZ-f/TZ-r were designed from the S RNAs of CCSV-TW (AY867502), TNSaV-2009-GZT (KM355773) and TZSV-Tomato-YN (NC_010489), respectively, using Primer3 (http://workbench.sdsc.edu/). The sequences of individual primers are listed in Table 1. One-step RT-PCR was conducted using a 25 μl of mixture consisting of 10 μg of total RNA extracted from virus-infected plant tissues, 100 nM individual primer pairs, 25 U One-Step RT-PCR enzyme mix, 1/5 volume of reaction buffer and 1/5 volume of enhancer buffer (GMbiolab). Samples were first incubated in 50 °C for 30 min for reverse transcription, then the PCR was performed with 35 cycles of strand separation at 94 °C for 30 s, annealing at 58 °C for 30 s and extension at 72 °C for 1 min, and a final reaction at 72 °C for 7 min. The PCR products were visualized by 1.2 % agarose gel electrophoresis.

**Table 1:** Species-specific primer pairs used for identification of individual tospoviruses

Virus	Primer name	Sequence (5′→3′)	Position at S RNA
Calla lily chlorotic spot virus (CCSV)	CC-f	GTGCTGCATAATGGAATTCAGCTG	2670–2693
	CC-r	GACTCTGGGATTCAATTTCAGT	2998–3019
Tomato necrotic spot associated virus (TNSaV)	TN-f	TGAGAGTAACGGGAGCGGACCACCT	2731–2755
	TN-r	AGGAAACAAGTGTTTGCTGCATG	3013–3035
Tomato zonate spot virus (TZSV)	TZ-f	GGCCATGCTGATAAGTCTAGTCCT	2889–2912
	TZ-r	ACCCAAGGCTTCAGCTTTGCCT	3104–3125

### Field sample collection

Between January and September of 2015, field tobacco (*Nicotiana tabacum* L.), tomato (*Solanum lycopersicum* L.), pepper (*Capsicum annuum* L.) and spider lily (*Crinum asiaticum* L.) samples showing tospovirus-like symptoms were collected from Zhaotong, Xishuangbanna, Kunming and Honghe in Yunnan Province, southwestern China to detect TZSV infections.

## Results

### Expression and purification of the TZSV rNP

To generate antibodies against the NP of TZSV, the full-length NP ORF of TZSV-13YV639 was expressed in *E. coli*, and its identity was verified by immunoblotting using RAs-His and MAb-CCSV-NP [25]. The resulting rNP was found mainly in the insoluble fractions of cell contents. The rNP of TZSV was isolated directly from the insoluble fraction, eluted from the polyacrylamide gel, and obtained an estimated 150 μg of purified rNP from 100 ml bacterial culture.

### Generation of mouse antibodies against rNP of TZSV

**Fig. 1:**
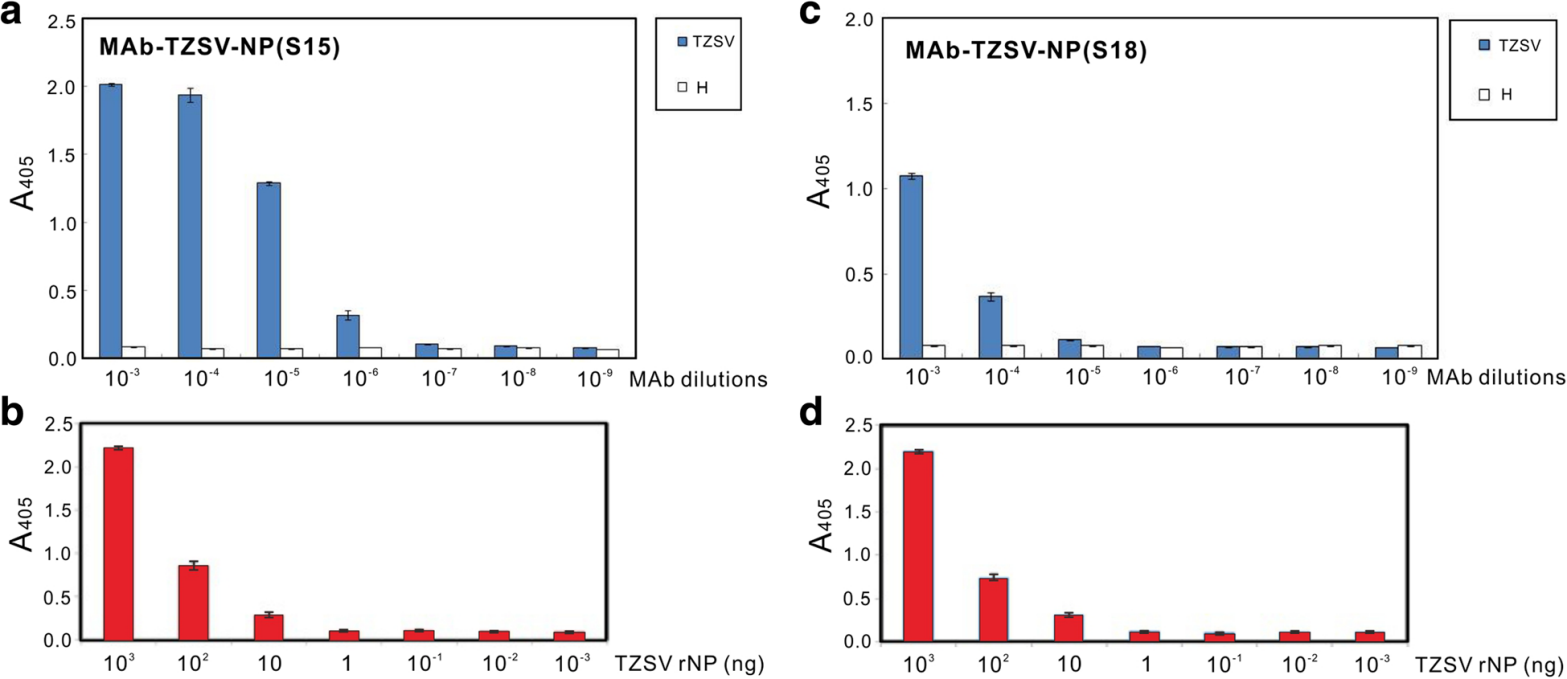
Titration assays of the produced monoclonal antibodies (MAbs). MAb-TZSV-NP(S15) (**a** and **b**) and MAb-TZSV-NP(S18) (**c** and **d**) against the nucleocapsid protein (NP) of Tomato zonate spot virus (TZSV) were assayed by indirect enzyme-linked immunosorbent assay. **a** and **c** the crude extracts from leaves of healthy (H, *white box*) and TZSV-13YV639-infected (*blue box*) *Nicotiana benthamiana* plants were used at a 1/50 dilution for testing the titers of the produced MAbs. The dilutions of MAbs are indicated at the X axis. **b** and **d** a 10-fold serial dilution of the purified bacterial-expressed recombinant NP (rNP, *red box*) of TZSV was used for analyzing the sensitivity of the produced MAbs. The amounts of rNP are indicated at the X axis. MAb-TZSV-NP(S15) was used at a 10^−4^ dilution and MAb-TZSV-NP(S18) was used at a 10^−3^ dilution for reacting with rNP. The absorbance at 405 nm (A_405_) recorded by a Model 680 microplate reader (Bio-Rad) at 30 min after colorization is shown at the Y axis

Two stable hybridoma lines S15D5H6 and S18D7H2 secreting antibodies against TZSV rNP were screened against crude sap of *N. benthamiana* tissues infected with TZSV-13YV639. The IgG-containing ascitic fluids produced from mice administered with S15D5H6 and S18D7H2 were designated as MAb-TZSV-NP(S15) and MAb-TZSV-NP(S18), respectively. The titer of MAb-TZSV-NP(S15) was determined as 10^−7^ by indirect ELISA (the average reading of TZSV-infected sample = 0.15 ± 0.001 compared with the healthy control = 0.069 ± 0.001), and the 10^−4^ dilution was used to detect at least 10 ng of purified rNP (Fig. 1a). The titer of MAb-TZSV-NP(S18) was determined as 10^−5^ by indirect ELISA (the average reading of TZSV-infected sample = 0.255 ± 0.03 compared with the healthy control = 0.064 ± 0.03), and the 10^−3^ dilution was used for further assays. At least 10 ng of purified rNP was detected by MAb-TZSV-NP(S18) at the 10^−3^ dilution (Fig. 1b).

### Serological reactions of the produced MAbs

**Fig. 2:**
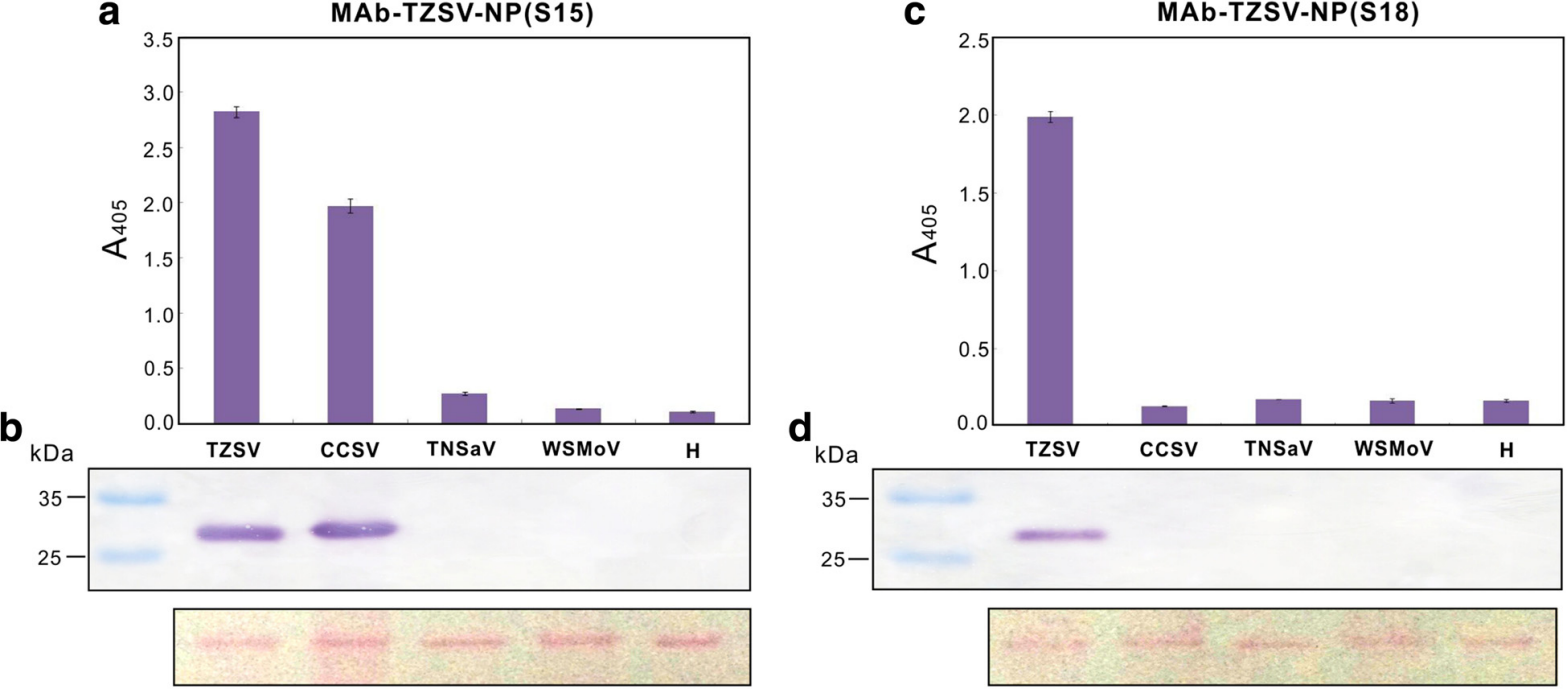
Analyses of the produced monoclonal antibodies reacted with Tomato zonate spot virus (TZSV) and other *Watermelon silver mottle virus* (WSMoV)-serogroup members. MAb-TZSV-NP(S15) (**a** and **b**) and MAb-TZSV-NP(S18) (**c** and **d**) were used at the 10^−4^ dilution and 10^−3^ dilution, respectively, in indirect enzyme-linked immunosorbent assay (**a** and **c**) and immunoblotting (**b** and **d**). The leaf crude extracts of *Nicotiana benthamiana* plants separately infected with Calla lily chlorotic spot virus (CCSV), Tomato necrotic spot associated virus (TNSaV), TZSV or WSMoV were used for serological assays. The crude extracts of a healthy *N. benthamiana* leaf (H) was used as the negative control. The plant ribulose bisphosphate carboxylase/oxygenase (rubisco) is shown by Ponceau S staining as a control in immunoblotting to indicate the loading quantity

**Fig. 3:**
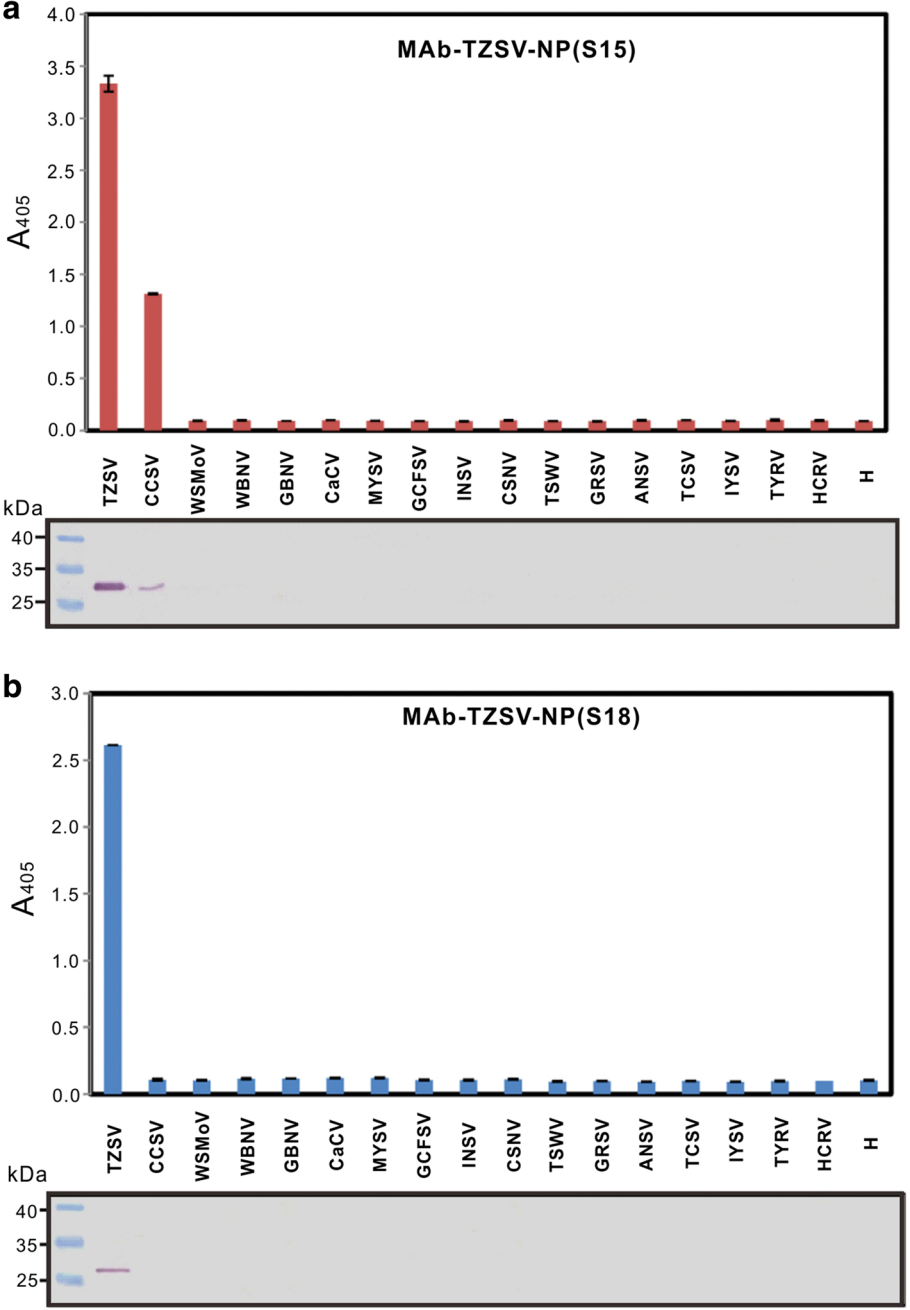
Serological reaction analyses of the produced monoclonal antibodies MAb-TZSV-NP(S15) (**a**) and MAb-TZSV-NP(S18) (**b**). The leaf crude extracts of *Nicotiana benthamiana* plants separately infected with Alstroemeria necrotic streak virus (ANSV), Calla lily chlorotic spot virus (CCSV), Capsicum chlorosis virus (CaCV), Chrysanthemum stem necrosis virus (CSNV), *Groundnut bud necrosis virus* (GBNV), Groundnut chlorotic fan-spot virus (GCFSV), *Groundnut ringspot virus* (GRSV), Hippeastrum chlorotic ringspot virus (HCRV), *Impatiens necrotic spot virus* (INSV), *Iris yellow spot virus* (IYSV), Melon yellow spot virus (MYSV), *Tomato chlorotic spot virus* (TCSV), *Tomato spotted wilt virus* (TSWV), Tomato yellow ring virus (TYRV), Tomato zonate spot virus (TZSV), *Watermelon bud necrosis virus* (WBNV) or *Watermelon silver mottle virus* (WSMoV) were used for indirect enzyme-linked immunosorbent assay (upper panels) and immunoblotting (lower panels). The crude extract of a healthy *N. benthamiana* leaf (H) was used as the negative control

**Fig. 4:**
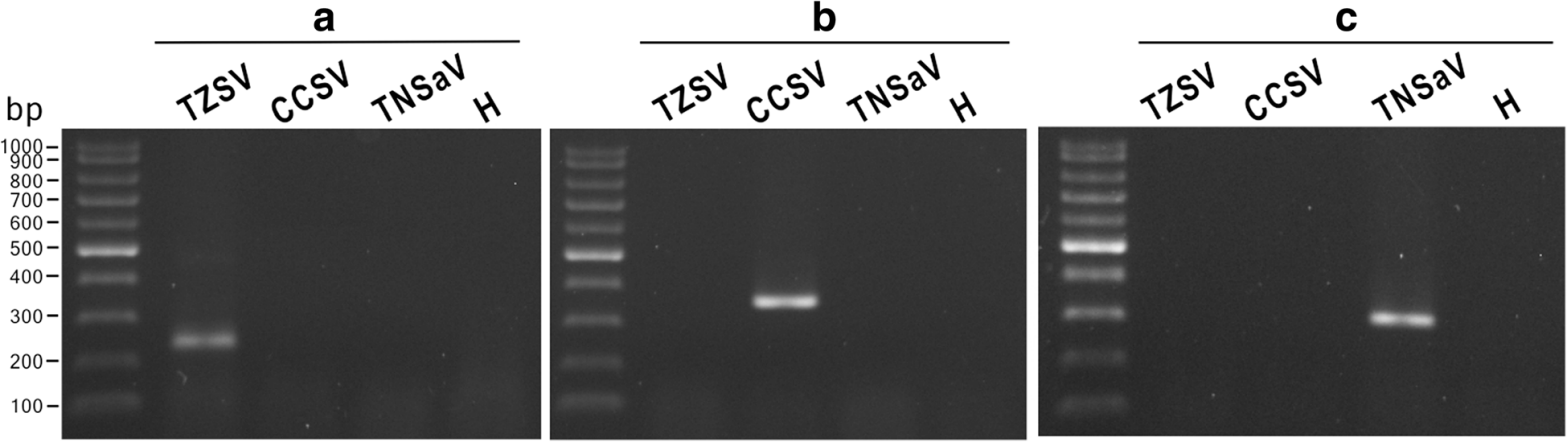
Identification of Calla lily chlorotic spot virus (CCSV), Tomato zonate spot virus (TZSV) and Tomato necrotic spot associated virus (TNSaV) by reverse transcription-polymerase chain reaction (RT-PCR). Total RNAs extracted from the virus-infected leaves of *Nicotiana benthamiana* plants were used for assays. Total RNA from healthy *N. benthamiana* plant (H) was used as a negative control. The TZSV-specific primer pair TZ-f/TZ-r (**a**), the CCSV-specific primer pair CC-f/CC-r (**b**) and the TNSaV-specific primer pair TN-f/TN-r (**c**) can be used to amplify 237, 350 and 305 bp of DNA fragments, respectively

Eighteen tospovirus species were used to evaluate the specificity of MAb-TZSV-NP(S15) and MAb-TZSV-NP(S18) in indirect ELISA and immunoblotting. MAb-TZSV-NP(S15) reacted with CCSV and homologous TZSV antigen but not with other tested tospoviruses, except a weak cross reactivity with TNSaV in indirect ELISA was found (Figs. 2a, b and 3a). MAb-TZSV-NP(S18) reacted with the homologous TZSV antigen only (Figs. 2c, d and 3b). The previously reported MAb-CCSV-NP [25] and MAb-WNSs [26] reacted with CCSV, TNSaV and TZSV (Additional file 2: Figure S1). The identity of the viruses that were detected by these antibodies was verified by RT-PCR using the primers specific to CCSV, TNSaV or TZSV. Amplicons were in the expected sizes for each virus species (237, 350 and 305 bp for TZSV, CCSV and TNSaV, respectively) (Fig. 4).

### Epitope mapping of MAb-TZSV-NP(S18)

**Fig. 5:**
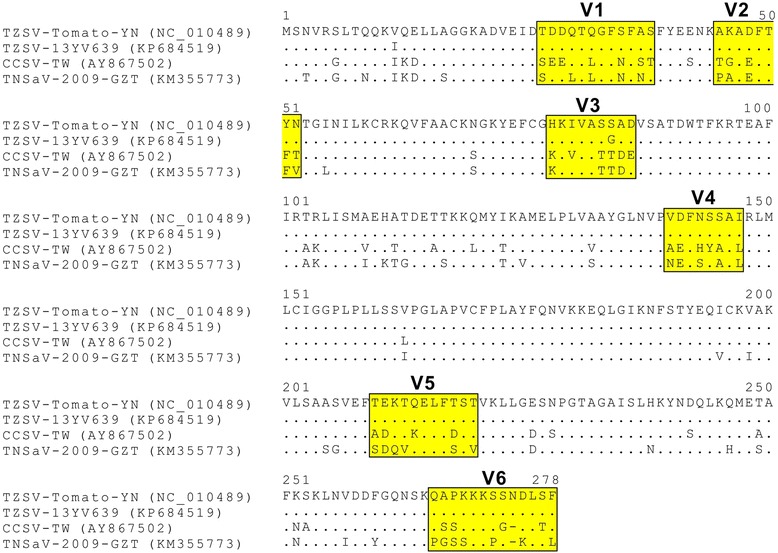
Multiple alignments of the nucleocapsid protein (NP) sequences of Calla lily chlorotic spot virus (CCSV), Tomato zonate spot virus (TZSV) and Tomato necrotic spot associated virus (TNSaV). The GenBank accession numbers of virus isolates used for comparison are indicated. The positions of variable regions (V1-V6) are highlighted by yellow boxes. The identical amino acid (aa) residues are represented by dots. The lacking aa residues are represented by hyphens

**Fig. 6:**
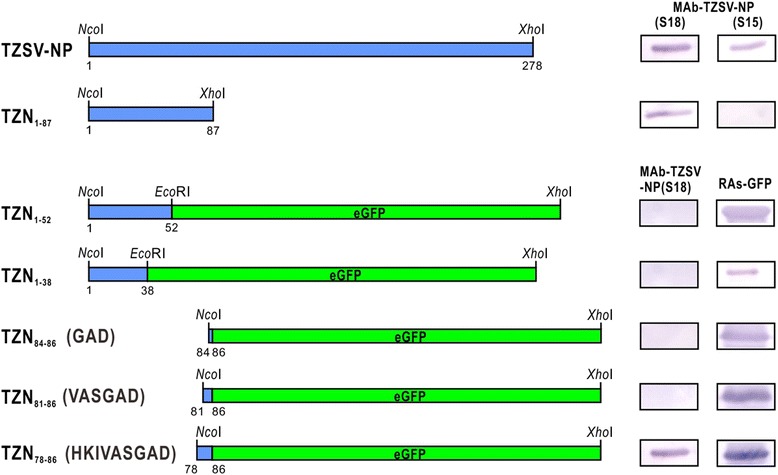
Schematic representation for epitope mapping of the monoclonal antibody MAb-TZSV-NP(S18). Immunoblotting was performed for assays. The produced MAb-TZSV-NP(S18) and MAb-TZSV-NP(S15) were used to react with the bacterial-expressed complete nucleocapsid protein (NP) open reading frame (ORF) of Tomato zonate spot virus (TZSV) and truncated TZN_1–87_. The truncated TZN_1–52_, TZN_1–38_, TZN_84–86_, TZN_81–86_ and TZN_78–86_ were expressed by fusing with enhanced green fluorescent protein (eGFP) and verified by the antiserum RAs-GFP [40]. The amino acid residues of TZN_84–86_, TZN_81–86_ and TZN_78–86_ are shown in parentheses. The expressed portions of TZSV NP are indicated by blue boxes. The eGFP ORF is indicated by green boxes. The restriction enzyme sites used for constructions are shown

The epitope of MAb-TZSV-NP(S18) was next characterized to better understand the protein regions unique to TZSV. Multiple alignments of the NPs of CCSV, TNSaV and TZSV showed that six variable regions are present at the positions of aa 27–38 (V1), aa 45–52 (V2), aa 78–86 (V3), aa 140–147 (V4), aa 210–218 (V5) and aa 266–278 (V6) of the TZSV NP (Fig. 5). The N-terminal aa 1–87 portion (TZN_1–87_) of the TZSV-13YV639 NP, which contains V1-V3, was expressed and reacted with MAb-TZSV-NP(S18) but not with MAb-TZSV-NP(S15). The portions of aa 1–38 (TZN_1–38_) and aa 1–52 (TZN_1–52_) of NP, containing V1 and V2 respectively, were expressed as a recombinant protein fused with eGFP, but did not observe any reactions with MAb-TZSV-NP(S18). These results suggest that the target site of MAb-TZSV-NP(S18) locates at the V3 region. The residues of V3, TZN_84–86_ (aa 84–86), TZN_81–86_ (aa 81–86) and TZN_78–86_ (aa 78–86), were further expressed at the N-terminal end of eGFP to assay for MAb binding. MAb-TZSV-NP(S18) detected the recombinant eGFP carrying TZN_78–86_, indicating that the residues of aa 78–86 (HKIVASGAD) is the deduced epitope of MAb-TZSV-NP(S18) (Fig. 6). The expression of all recombinant eGFPs was validated by also detecting the proteins using antibodies against GFP (RAs-GFP) [40].

### Application of the TZSV MAbs in virus detection on filed samples

**Fig. 7:**
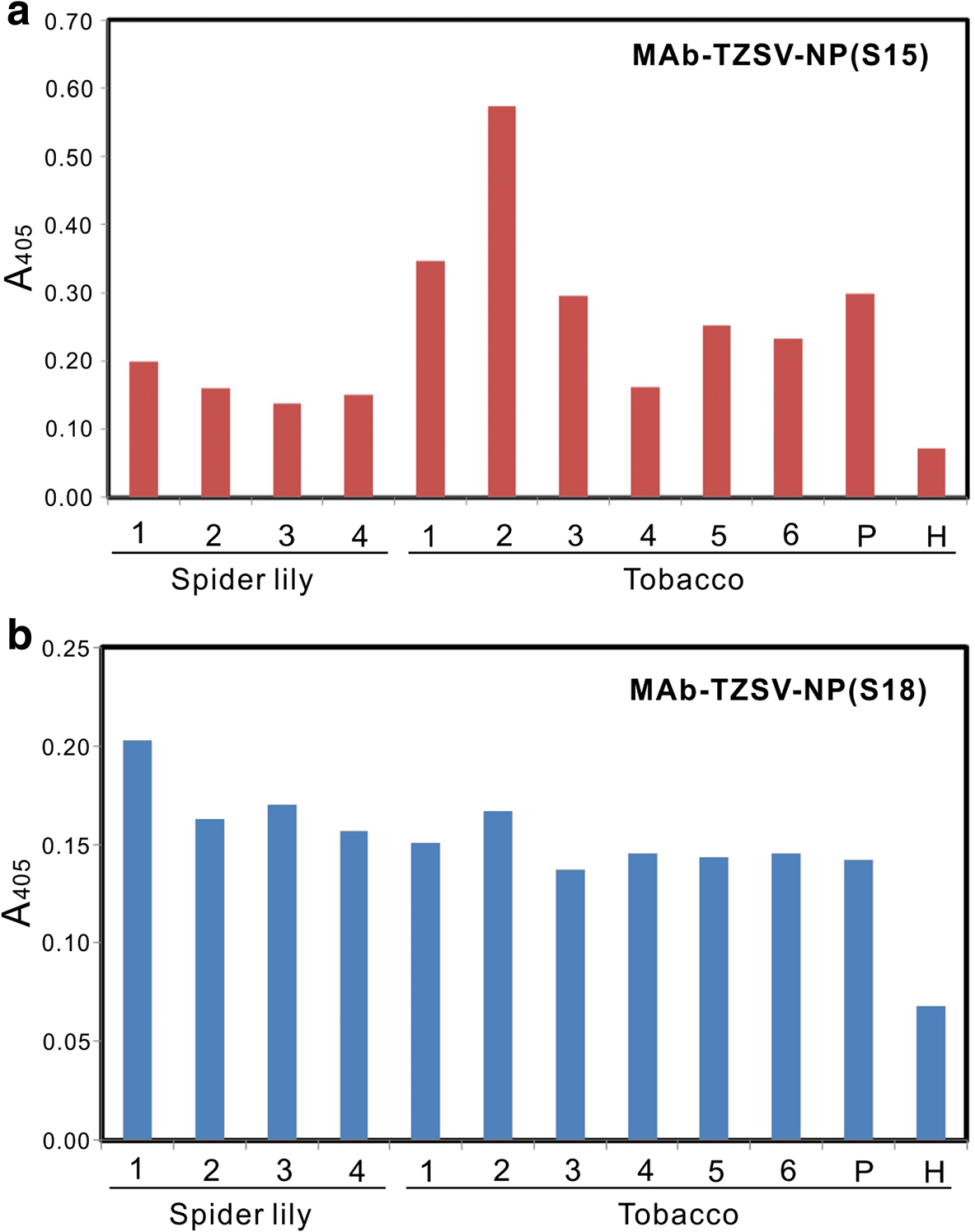
Reaction of the produced monoclonal antibodies (MAbs) with different Tomato zonate spot virus (TZSV) isolates. Four TZSV isolates from spider lily (*Crinum asiaticum*) and six isolates from tobacco (*Nicotiana tabacum*) collected from fields in Yunnan, China were used to react with MAb-TZSV-NP(S15) (**a**) and MAb-TZSV-NP(S18) (**b**) in indirect enzyme-linked immunosorbent assay. The crude extract of tobacco plant infected with the original TZSV Tomato-YN isolate was used as the positive control (P). The crude extract of a healthy tobacco plant (H) was used as the negative control. Positive reaction was judged by the mean reading is twice higher than the mean reading of the negative control

Both MAb-TZSV-NP(S15) and MAb-TZSV-NP(S18) were successfully used to react with the original Tomato-YN isolate [20] and other TZSV isolates collected from spider lily and tobacco, which were preserved by Yunnan Provincial Key Laboratory of Agricultural Biotechnology, Yunnan Academy of Agricultural Sciences, by indirect ELISA (Fig. 7). The detection efficiency of TZSV between the produced MAbs was compared in a field survey in 2015. A total of 187 diseased field samples of pepper, spider lily, tobacco and tomato collected from Honghe, Kunming, Xishuangbanna and Zhaotong of Yunnan Province were used for assays by indirect ELISA. Results showed that 60 (32.1 %) and 48 (25.7 %) samples were tested for TZSV positive by MAb-TZSV-NP(S15) and MAb-TZSV-NP(S18), respectively (Table 2).

**Table 2:** Field survey for Tomato zonate spot virus (TZSV) using the produced monoclonal antibodies MAb-TZSV-NP(S15) and MAb-TZSV-NP(S18)

Plant	Total sample no.	MAb-TZSV-NP(S15)-positive no.	MAb-TZSV-NP(S18)-positive no.	Location
Tobacco (*Nicotiana tabacum*)	114	34	26	Honghe
Tomato (*Solanum lycopersicum*)	40	13	9	Zhaotong, Honghe and Kunming
Pepper (*Capsicum annuum*)	25	9	9	Zhaotong, Honghe and Kunming
Spider lily (*Crinum asiaticum*)	8	4	4	Kunming and Xishuangbanna
Sum	187	60	48	

## Discussion

There is a need to develop tools to identify and diagnose tospoviruses in the field earlier to prevent a disease from becoming an epidemic. In eastern Asian countries, including mainland China, India, Japan, Taiwan and Thailand, tospoviruses cause severe agricultural problems. Most of tospoviruses known to prevail in these countries are clustered in the WSMoV serogroup that can be detected by serological assays using the group-broad antiserum RAs-WSMoV-NP against the WSMoV NP [13] and MAb-WNSs produced from the NSs protein of WSMoV [26]. The NP MAbs are useful for differentiation of most tospovirus species in a serogroup [13, 25]. However, the tospovirus species sharing over 80 % NP amino acid identity are still difficult to distinguish even when MAbs were used [17], and an additional RT-PCR analysis using species-specific primers is required.

CCSV, TNSaV and TZSV also share higher than 80 % NP amino acid identity with each other [19, 20]. Their close serological relationship was experimentally demonstrated by reacting with MAb-WNSs and MAb-CCSV-NP in both indirect ELISA and immunoblotting (Additional file 2: Figure S1). Here, we attempted to produce MAbs specific to TZSV for developing a method to specifically diagnose each virus without the need of additional RT-PCR analysis. Eighteen tospovirus species representing the four major serogroups were used to test the serological reaction of the two prepared MAbs. Moreover, the identity of viruses was verified by RT-PCR analyses using the newly designed primer pairs specific to CCSV, TNSaV or TZSV. The reaction of the obtained MAb-TZSV-NP(S15) supports a closer serological relatedness between TZSV and CCSV rather than TNSaV. We noticed a weak reaction with a two-fold mean reading (0.27) higher than those of the negative controls WSMoV-infected (0.13) and healthy (0.11) plants when MAb-TZSV-NP(S15) incubated with TNSaV in indirect ELISA (Fig. 2a), but no signal was found in immunoblotting (Fig. 2b). The loading and transfer of proteins were confirmed by Ponceau S staining, therefore it is unlikely that this resulted from an experimental error, and suggests that the protein region recognized by MAb-TZSV-NP(S15) is conserved in CCSV and TZSV, but not in the NP of TNSaV. According to the results of the epitope assay of MAb-TZSV-NP(S18) and multiple alignments of NPs, the epitope of MAb-TZSV-NP(S15) could be near the V5 and V6 regions of the TZSV NP, in which shares higher homology between CCSV and TZSV than TNSaV (Fig. 5). The antigenic epitope of MAb-TZSV-NP(S15) needs to be further characterized.

In contrast, MAb-TZSV-NP(S18) is TZSV-specific. The deduced antigenic epitope of MAb-TZSV-NP(S18) is aa 78–86 region of TZSV NP, which is unique to TZSV. Most residues of aa 78–86 are conserved in the NPs of the reported TZSV isolates, except the aa-84 residue that is glycine in the TZSV-13YV639 isolate used in this study or serine in the original TZSV-Tomato-YN isolate [20]. Serological results showed that MAb-TZSV-NP(S18), as well as MAb-TZSV-NP(S15), can be used to react with different TZSV isolates collected from fields in Yunnan. This includes the Tomato-YN isolate (Fig. 7) with the difference in aa-84 residue, and suggests that the MAb-TZSV-NP(S18) is a valuable tool for detecting TZSV in field surveys. We detected higher tospovirus infections when MAb-TZSV-NP(S15) was used (Table 2), which may have resulted from the higher titer of MAb-TZSV-NP(S15). The presence of other tospoviruses serologically related with TZSV, such as CCSV and TNSaV, could be excluded from the collected field samples by RT-PCR.

Some antibodies have higher titer due to the nature of the epitope. When the aa sequence of TZSV-13YV639 NP is used to predict antibody epitope using the B cell epitope prediction tool of IEDB Analysis Resource (http://tools.immuneepitope.org/bcell), the results suggested that part of the MAb-TZSV-NP(S18) epitope (aa 84–86) conforms with the result of antibody epitope prediction. Several epitopes can be predicted in the aa 88–278 region of TZSV NP with higher scores than that of aa 78–86. The titer difference between MAb-TZSV-NP(S18) and MAb-TZSV-NP(S15) could have resulted from the B cell-targeting property of antigenic epitopes. The epitope of MAb-TZSV-NP(S15) is likely to be in aa 88–278 position of the NP, and the IEDB tool predicts a higher score compared to the epitope of MAb-TZSV-NP(S18).

Both MAb-TZSV-NP(S15) and MAb-TZSV-NP(S18) are successfully used to detect TZSV in natural diseased plant samples. This is the first report to show that approximately 30 % TZSV incidence can be detected in a one-year field survey. Since TZSV was first reported in 2008, several TZSV isolates have been identified in numerous crops in Yunnan Province by RT-PCR [20–22]. Actually, the detection of TZSV in field plant samples was also conducted by ELISA using the antiserum against the TZSV NP, which was also used to react with TNSaV [19]. Taken together with our previous results and the field survey conducted in 2015, we indicate that TZSV is prevailing in Yunnan Province infecting numerous important economic crops, such as pepper, tobacco and tomato, and the ornamental spider lily.

Although CCSV, TNSaV and TZSV all occur in mainland China, only CCSV has been found in Taiwan [19, 20, 23, 24]. The quarantine of imported agricultural products for TNSaV and TZSV is important to prevent their invasion in Taiwan. We proposed an efficient serological detection platform for virus inspection that MAb-CCSV-NP [25] is used to detect all CCSV, TNSaV and TZSV; MAb-TZSV-NP(S15) is used to exclude TNSaV from TZSV and CCSV; and MAb-TZSV-NP(S18) is used to identify TZSV (Additional file 3: Table S2). This assay should provide a method that relies on protein analysis only, and will improve the speed at which tospovirus infections can be detected.

## Conclusions

In this study, the close serological relatedness of CCSV, TNSaV and TZSV clustered in the WSMoV serogroup is experimentally demonstrated from the cross reaction with the previous reported MAb-CCSV-NP [25] and MAb-WNSs [26]. Two new MAbs against the TZSV NP, MAb-TZSV-NP(S15) and MAb-TZSV-NP(S18), with distinct serological reactivity were obtained. Epitope mapping analyses revealed that the MAb-TZSV-NP(S18) targets a highly conserved region, the residues of aa _78_HKIVASGAD_86_, at the NPs of known TZSV isolates that is highly specific and suitable for identifying the TZSV species. MAb-TZSV-NP(S15) reacting with both CCSV and TZSV can be used to exclude TNSaV. The TZSV MAbs were applied in field survey in 2015, showing that TZSV is prevailing on economic crops including pepper, tobacco and tomato and the ornamental spider lily in Yunnan Province. All MAb-CCSV-NP, MAb-TZSV-NP(S15) and MAb-TZSV-NP(S18) do not react with other tested tospoviruses ANSV, CaCV, CSNV, GBNV, GCFSV, GRSV, HCRV, INSV, IYSV, MYSV, TCSV, TSWV, TYRV, WBNV and WSMoV. Here we proposed a serological detection platform using these three MAbs to allow researchers and quarantine staff to efficiently diagnose the infections of CCSV, TNSaV and TZSV in China and other countries.

## Additional files

Additional file 1: Table S1. Primers used for amplification of complete or truncated open reading frames of nucleocapsid (N) gene of Tomato zonate spot virus (TZSV) 13YV639 isolate. (DOCX 14 kb) Additional file 2: Figure S1. Comparison of the serological relationship of Calla lily chlorotic spot virus (CCSV), Tomato zonate spot virus (TZSV) and Tomato necrotic spot associated virus (TNSaV). The previously reported monoclonal antibodies MAb-CCSV-NP [24] (**A** and **B**) and MAb-WNSs [25] (**C** and **D**) were used to react with the crude leaf extracts of *Nicotiana benthamiana* plants separately infected with CCSV, TZSV, TNSaV or *Watermelon silver mottle virus* (WSMoV) in indirect enzyme-linked immunosorbent assay (**A** and **C**) and immunoblotting (**B** and **D**). The crude extract of a healthy *N. benthamiana* leaf (H) was used as the negative control. The plant ribulose bisphosphate carboxylase/oxygenase (rubisco) of Ponceau S staining is shown under immunoblotting to indicate the loading quantity. MAb-CCSV-NP was used at a 10^−4^ dilution. MAb-WNSs was used at a 10^−3^ dilution. (DOCX 124 kb) Additional file 3: Table S2. Serological platform using three monoclonal antibodies for identification of Calla lily chlorotic spot virus (CCSV), Tomato zonate spot virus (TZSV) and Tomato necrotic spot associated virus (TNSaV). (DOCX 12 kb)

## Abbreviations

ANSV: Alstroemeria necrotic streak virus
CCSV: Calla lily chlorotic spot virus
CaCV: Capsicum chlorosis virus
CSNV: Chrysanthemum stem necrosis virus
ELISA: enzyme-linked immunosorbent assay
GBNV: *Groundnut bud necrosis virus*
GCFSV: Groundnut chlorotic fan-spot virus
GRSV: *Groundnut ringspot virus*
GYSV: *Groundnut yellow spot virus*
HCRV: Hippeastrum chlorotic ringspot virus
INSV: *Impatiens necrotic spot virus*
IYSV: *Iris yellow spot virus*
MYSV: Melon yellow spot virus
MAb: Monoclonal antibody
NP: nucleocapsid protein
ORF: Open reading frame
RT-PCR: reverse transcription-polymerase chain reaction
TCSV: *Tomato chlorotic spot virus*
TNSaV: Tomato necrotic spot associated virus
TSWV: *Tomato spotted wilt virus*
TYRV: Tomato yellow ring virus
TZSV: Tomato zonate spot virus
WBNV: *Watermelon bud necrosis virus*
WSMoV: *Watermelon silver mottle virus*
