# Supplementary material for: Monoclonal antibodies for differentiating infections of three serological-related tospoviruses prevalent in Southwestern China
Source: Virol J. 2016 Apr 27;13:72. doi: 10.1186/s12985-016-0525-3 (PMC4848788; doi:10.1186/s12985-016-0525-3)
Supplement: Additional file 1: Table S1. — Primers used for amplification of complete or truncated open reading frames of nucleocapsid (N) gene of Tomato zonate spot virus (TZSV) 13YV639 isolate. (DOCX 14 kb) [file 12985_2016_525_MOESM1_ESM.docx]

**Table S1.** Primers used for amplification of complete or truncated open reading frames of nucleocapsid (N) gene of Tomato zonate spot virus (TZSV) 13YV639 isolate

| Primer name | Sequence (5'→3') | Restriction enzyme site (underlined) | Position at N gene (italic) |
| --- | --- | --- | --- |
| Forward |  |  |  |
| TZN-NcoI | GCC*ATGGCTAACGTCCGGAGTTTAACAC* | *Nco*I | 1-25 |
| TZN-NcoI-84-86aa | GCCATG*GGTGCTGAT*GTGAGCAAGGGCGAGGAGCTGTTCACCGGG | *Nco*I | 250-258 |
| TZN-NcoI-81-86aa | GCCATG*GTTGCTAGTGGTGCTGAT*GTGAGCAAGGGCGAGGAGCTG | *Nco*I | 241-258 |
| TZN-NcoI-77-86aa | GCCATG*GGTCATAAAATTGTTGCTAGTGGTGCTGAT*GTGAGCAAGGGC | *Nco*I | 229-258 |
| Reverse |  |  |  |
| TZN-Xho-Kpnc | GCTCGAGTTAGGTACC*AAAAGACAGATCATTGCTGCTCTTCTT* | *Xho*I | 808-834 |
| TZN-EcoRI-38aa | GGAATTC*AGAGGCAAAGCTGAAGCCTTG* | *Eco*RI | 94-114 |
| TZN-EcoRI-52aa | GGAATTC*GTTGTAAGTAAAATCAGCTTT* | *Eco*RI | 136-156 |
| TZN-Xho-87aa | GCTCGAG*CACATCAGCACCACTAGCAAC* | *Xho*I | 241-261 |
