# Supplementary material for: Monoclonal antibodies for differentiating infections of three serological-related tospoviruses prevalent in Southwestern China
Source: Virol J. 2016 Apr 27;13:72. doi: 10.1186/s12985-016-0525-3 (PMC4848788; doi:10.1186/s12985-016-0525-3)
Supplement: Additional file 2: Figure S1. — Comparison of the serological relationship of Calla lily chlorotic spot virus (CCSV), Tomato zonate spot virus (TZSV) and Tomato necrotic spot associated virus (TNSaV). The previously reported monoclonal antibodies MAb-CCSV-NP [24] (A and B) and MAb-WNSs [25] (C and D) were used to react with the crude leaf extracts of Nicotiana benthamiana plants separately infected with CCSV, TZSV, TNSaV or Watermelon silver mottle virus (WSMoV) in indirect enzyme-linked immunosorbent assay (A and C) and immunoblotting (B and D). The crude extract of a healthy N. benthamiana leaf (H) was used as the negative control. The plant ribulose bisphosphate carboxylase/oxygenase (rubisco) of Ponceau S staining is shown under immunoblotting to indicate the loading quantity. MAb-CCSV-NP was used at a 10−4 dilution. MAb-WNSs was used at a 10−3 dilution. (DOCX 124 kb) [file 12985_2016_525_MOESM2_ESM.docx]

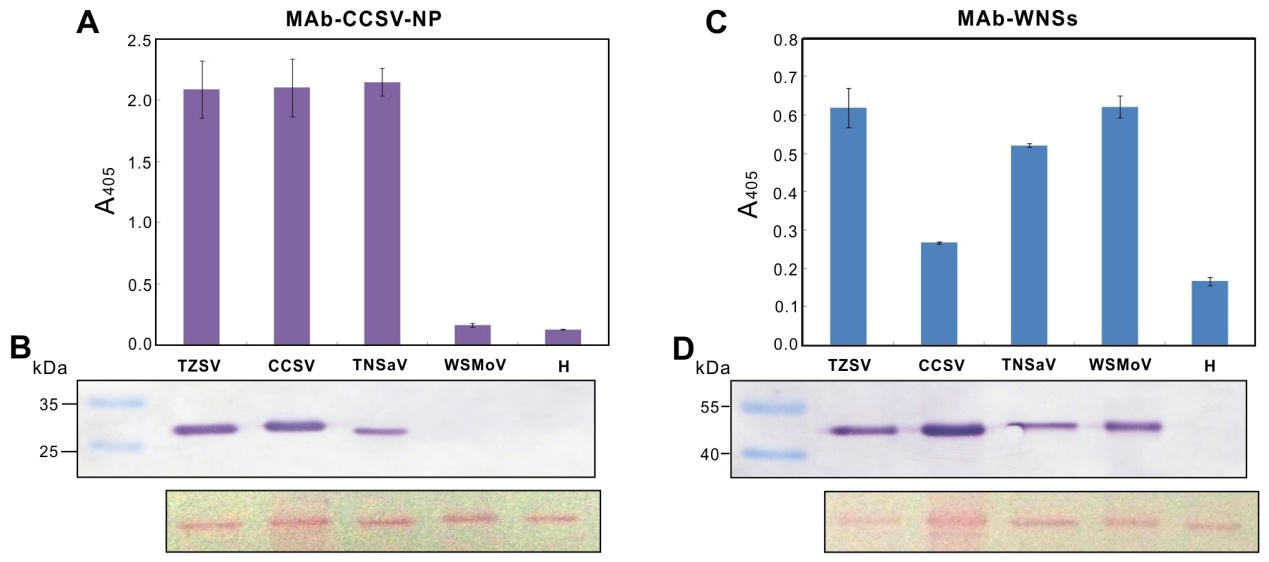


**Figure S1.** Comparison of the serological relationship of Calla lily chlorotic spot virus (CCSV), Tomato zonate spot virus (TZSV) and Tomato necrotic spot associated virus (TNSaV). The previously reported monoclonal antibodies MAb-CCSV-NP [24] (**A and B**) and MAb-WNSs [25] (**C and D**) were used to react with the crude leaf extracts of *Nicotiana benthamiana* plants separately infected with CCSV, TZSV, TNSaV or *Watermelon silver mottle virus* (WSMoV) in indirect enzyme-linked immunosorbent assay (**A and C**) and immunoblotting (**B and D**). The crude extract of a healthy *N. benthamiana* leaf (H) was used as the negative control. The plant ribulose bisphosphate carboxylase/oxygenase (rubisco) of Ponceau S staining is shown under immunoblotting to indicate the loading quantity. MAb-CCSV-NP was used at a 10^-4^ dilution. MAb-WNSs was used at a 10^-3^ dilution.
