# Supplementary material for: Monoclonal antibodies for differentiating infections of three serological-related tospoviruses prevalent in Southwestern China
Source: Virol J. 2016 Apr 27;13:72. doi: 10.1186/s12985-016-0525-3 (PMC4848788; doi:10.1186/s12985-016-0525-3)
Supplement: Additional file 3: Table S2. — Serological platform using three monoclonal antibodies for identification of Calla lily chlorotic spot virus (CCSV), Tomato zonate spot virus (TZSV) and Tomato necrotic spot associated virus (TNSaV). (DOCX 12 kb) [file 12985_2016_525_MOESM3_ESM.docx]

**Table S2.** Serological platform using three monoclonal antibodies for identification of Calla lily chlorotic spot virus (CCSV), Tomato zonate spot virus (TZSV) and Tomato necrotic spot associated virus (TNSaV)

|  | **MAb-CCSV-NP** | **MAb-TZSV-NP(S15)** | **MAb-TZSV-NP(S18)** |
| --- | --- | --- | --- |
| **TZSV** | **+** | **+** | **+** |
| **CCSV** | **+** | **+** | **-** |
| **TNSaV** | **+** | **-** | **-** |
